# An Explanation

**Published:** 1868-11

**Authors:** 


*An Explanation.*--Several years ago it was our good fortune to observe the manipulation of a form of crystal gold prepared by Dr. G. Watt, and ever since we have been at a loss to account for it non-appearance at the dental depots. The following editorial of Prof. Watt, on "Plastic Gold," in the Oct. No. of the *Dental Register*, explains the matter!

"The effort made by members of our profession and others to obtain a reliable variety of plastic gold for the market have not yet proved entirely successful. Many difficulties have arisen; and some of these have been overcome. None of them afford the slightest argument against the use of plastic gold for filling teeth, when properly prepared and legitimately used. Crystallized gold has been prepared in plastic masses, so as to make perfect fillings, with less labor, and in less time than are necessary to produce like results with foil. Fillings from such material, inserted in 1853, with badly shaped, unserrated instruments, before the operators had learned the peculiar manipulations necessary to the best possible results, are now solid masses of gold, saving and *strengthening* the teeth which contain them. This too, notwithstanding that the profession had as yet failed to recognize the *welding* properties of gold. (When the writer of this, in 1856, in the American Dental Convention, stated that, "gold is preeminently *the* welding metal, being the only one that can be welded at ordinary temperatures, his statement was met by doubt, and even treated with derision.)

A few thoughts on the causes of failure, and if able, a few hints that may lead to useful results, are all that will be aimed at now. It is well known that the crystal gold effort has been gravely pronounced a failure *in toto;* but, in view of the facts above referred to, which are by no means lonely, it would be as consistent to deny that coition and conceptions are essential to the propagation of the race, because some of us raise no babies.

Before me is a small mass of plastic gold. The microscope reveals that it is definitely crystallized. Pressure reveals that it is plastic. Pressed on a bar of steel whose surfaceis indented with pits invisible by the unassisted eye, the magnifier shows, on the gold, prominences counterpart to the pits. It is sufficiently plastic for practical use. It is at least four times as heavy as the same bulk of any plastic gold in the market. It is as *plastic* as any of them, though not as compressible. Now, if any of the plastic gold in the market is compressed one-fourth in its present bulk, it is far less plastic, and not nearly so compressible as this; and yet it has the same distance to travel—the same changes to undergo, in becoming solid gold that this has. This being true, we know that the plastic golds, as now furnished, are all too porus. Many speak of the *plasticity* of gold when they really refer to its *porosity*.

The plastic golds in the market are simply precipitates. Varieties may be obtained in several ways. The strength of the solution, the character of precipitant, the degree of rapidity with which it is added to the solution, the presence or absence of foreign matter, are some of the modifying circumstances. The electric condition of the atmosphere has probably something to do in preventing uniformity of results. At any rate, there is general complaint on this point. Members of the profession teil us they have been delighted with one specimen, and disgusted with the next from the same manufacturer. Manufacturers have repeatedly promised uniform results, honestly expecting to fulfil their promises, and have as often failed.

A part of the difficulty has risen from the fact that the manufacturers of the gold have not been practicing dentists. They could not know so well what was wanted; and not realizing the difficulties and defects, were less likely to labor for their removal. There is, perhaps some change in this respect recently.

The specimen before us was prepared by a modification of A. J. Watts' first patent—that is, as directeted by the writer in the *Dental Cosmos* a few years ago. There is nothing like it in the market. I do not think it can be produced by following the patent closely or blindly. It would sell like cigars, especially in the West—indeed in every place, unless in Philadelphia, where they gave a bur-drill trial to one of its comrades, and condemed the whole family. But this variety can not be afforded at the same prices as the precipitated golds or foils. But it would sell at prices that would justify its manufacture.

Many ask why it is not in the market; and like myself, many wish to buy it. No one who reads *Taft's Operative Dentistry* will believe he would use much foil if this gold could be purchased. I have tried to have it in the market. A number of times I have sent specimens of it to Dr. A. J. Watts by mail, and twice by the hands of professed friends, requesting him to furnish such an article for sale, assuring him it would sell much more rapidly in the West than that he was furnishing. I never heard either from him or the gold. Twice I tried to obtain an interview with him but failed. Those apparently co-operating with him seemed unwilling that I should see him; and from this I concluded that he never received either my communications or the gold. And I cannot object; for I was, no doubt, regarded as a spy, from the fact that Dr. Taft and I once prepared gold for sale. At the time we did so, I had no idea we were infringing on A. J. W's patent. As soon as I had a doubt on the subject I ceased to make the gold for sale; but tried to save it to the profession in the manner described; and regard it as quite unfortunate that I did not have the opportunity to confer with Dr. Watts. I do not believe an expert can make a *useful* preparation of gold by *following the patent;* hence, I doubt its validity, but have been disposed to respect it. But by modifying the process a far better article than is now for sale can be obtained.

A frank statement is thus made because I do not wish to be longer misunderstood. Inasmuch as this gold is confined to the offices of Watts and Williams. in Cincinnati and Xenia, we have been charged with selfishness, and suspected of unprofessional conduct, by some not duly informed of the facts, and possibly a little envious. We'd like to see that gold in the market."

